# Cnidarian Cell Cryopreservation: A Powerful Tool for Cultivation and Functional Assays

**DOI:** 10.3390/cells9122541

**Published:** 2020-11-26

**Authors:** Clara Fricano, Eric Röttinger, Paola Furla, Stéphanie Barnay-Verdier

**Affiliations:** 1 CNRS, INSERM, Institute for Research on Cancer and Aging (IRCAN), Université Côte d’Azur, 28 avenue de Valombrose, F-06107 Nice, France; clara.fricano@unice.fr (C.F.); eric.rottinger@unice.fr (E.R.); paola.furla@unice.fr (P.F.); 2 Sorbonne Université, UFR 927, 4 Place Jussieu, F-75252 Paris, France

**Keywords:** primary cell culture, sea anemone, *Anemonia viridis*, dimethyl sulfoxide (DMSO), marine invertebrate, post-thaw recovery

## Abstract

Cnidarian primary cell cultures have a strong potential to become a universal tool to assess stress-response mechanisms at the cellular level. However, primary cell cultures are time-consuming regarding their establishment and maintenance. Cryopreservation is a commonly used approach to provide stable cell stocks for experiments, but it is yet to be established for Cnidarian cell cultures. The aim of this study was therefore to design a cryopreservation protocol for primary cell cultures of the Cnidarian *Anemonia viridis*, using dimethyl sulfoxide (DMSO) as a cryoprotectant, enriched or not with fetal bovine serum (FBS). We determined that DMSO 5% with 25% FBS was an efficient cryosolution, resulting in 70% of post-thaw cell survival. The success of this protocol was first confirmed by a constant post-thaw survival independently of the cell culture age (up to 45 days old) and the storage period (up to 87 days). Finally, cryopreserved cells displayed a long-term recovery with a maintenance of the primary cell culture parameters and cellular functions: formation of cell aggregates, high viability and constant cell growth, and unchanged intrinsic resistance to hyperthermal stress. These results will further bring new opportunities for the scientific community interested in molecular, cellular, and biochemical aspects of cnidarian biology.

## 1. Introduction

In vitro cell cultures are important tools for research in many fields, including development, virology, cancer research, toxicity testing, biotechnology, and biomedicine, as well as for environmental research [1,2,3]. Mammalian cells lines are well established and commonly used since decades, followed by other vertebrates (e.g., zebrafish; for review Vallone et al., 2007 [4]) and insect cell lines (for review Lynn, 2001 [5]). Despite much effort devoted since the 1970s (see for reviews Rinkevich, 2005 [6], 2011 [7], and Cai and Zhang, 2014 [8]) marine invertebrate cell cultures are not as advanced. While marine invertebrate cell lines (i.e., permanently established cell cultures) are yet to be available, recent reports on the establishment of primary cell cultures are encouraging with maintenance and/or growing from a couple of days to weeks [9,10,11,12,13,14,15].

Once protocols for reproducibly initiating primary cell cultures are established, the next important obstacle to overcome is the development of preservation procedures in order to outreach primary cell cultures limitations, notably their limited lifespan. Indeed, such a preservation tool will reduce the frequency of primary cell culture establishment and will minimize wastage of a valuable resource at reseedings by creating cell stocks for as long as a primary cell culture is healthy. Among preservation procedures, cryopreservation is considered to be the optimal long-term storage method for maintaining a variety of biological materials, including cell cultures, in a state of metabolic arrest for considerable periods of time [16].

To date, for marine invertebrates, spermatozoa, oocytes, embryos, and different larval stages have been successfully cryopreserved mostly from Mollusk and Echinoderm species, but also from Arthropod and Cnidarian species (see for reviews Odintsova and Boroda, 2012 [17] and Paredes, 2015 [18]). Other biomaterials have been studied in a cryopreservation context, such as coral fragments [19] and primmorphs from sponge cells [20]. The tolerance to various cryoprotectants of tissue balls from corals was also investigated by Feuillassier et al. (2015) [21]. However, considering the limited advancements in marine invertebrate cell cultures, cryopreservation of marine invertebrate dissociated cells is seldom reported [18]. If they are, they largely focus on Mollusks [22,23,24,25,26,27,28,29,30], with some other studies conducted on Echinoderms (e.g., dissociated cells from sea urchins larvae [24,28]), on sponge cells [31,32,33,34] and on coral cells dissociated from embryos or larvae [35,36]. In addition, none of these studies, except those on sponge cells, maintained cryopreserved cells in cultures for more than a few days nor use cryopreserved cells for subsequent experiments.

One of the major factors that determines the success of a cryopreservation protocol is the type of cryoprotecting agents (CPAs) used [16]. CPAs prevent damage to the cells from changes in osmotic pressure and intracellular ice crystal formation. Among the various CPAs, dimethyl sulfoxide (DMSO), a penetrating CPA, is the most common and widely used cryoprotectant to maintain frozen cell lines. The precise mechanism by which it protects cells remains unclear; it has been suggested that DMSO depresses the freezing point of cryosolutions [37], and that it can modulate the water network hydrating the membrane hence reducing the stress induced by the volume changes of water during freeze-thaw [38]. Penetrating CPAs could, however, induce some cytotoxicity due to the disruption of intracellular signaling which results in cell death [39,40,41]. For marine invertebrate studies, Paredes (2015) [18] reported in her review that DMSO was the most effective CPA for 70% of the published work on germ cells, embryos and larvae compared to others CPAs such as glycerol. This trend is also found for marine invertebrate dissociated cells [21,22,33,35]. In addition, in marine invertebrate studies, DMSO was found as an efficient CPA on its own [22,29,35] but more frequently in combination with other CPAs or with proteins, vitamins or sugar cocktails [18,24,26,27,33]. Indeed, the preservative capacity of DMSO was long known to be increased when serum, such as fetal bovine serum (containing cocktail of proteins), is added to the cryosolution [42,43].

We have previously reported the establishment of primary cell cultures of a soft-body cnidarian, the temperate sea anemone *Anemonia viridis* [9]. The established cell culture protocol resulted in the maintenance of primary cell cultures with gastrodermal signature [15]. These cell cultures were successfully used to assess the cellular response (e.g., viability) to environmental stress [15] thus creating new perspectives for further fundamental, environmental and biotechnological questions. An efficient cryopreservation procedure would therefore be an essential and powerful tool for facilitating research in deciphering molecular mechanisms and cellular events in cnidarian cells.

The aim of this study was therefore to design a cryopreservation protocol for primary gastrodermal *A. viridis* cell cultures in order to ensure a high post-thaw cell survival, preserving long-term recovery: cell viability, cell growth, and physiological responses. All these advances will participate to raise the cnidarian cell cultures as a model system for marine invertebrate research perspectives.

## 2. Material and Methods

### 2.1. Biological Material

Five individuals of *Anemonia viridis* (Forskal 1775) were collected (prefectural authorization n°107; 28 February 2019) from ‘Plage des ondes’, Antibes, France, (43°33′17″ N, 7°07′17.7″ E), and maintained in a closed-circuit aquarium with artificial seawater (ASW) at 36–38‰ with Prodibio Expert Reef Salt, at 18.0 ± 0.5 °C with weekly water changes. An LED bar (450 nm—Deckey LED aquarium) provided light at a constant saturating irradiance of 100 μmol m^−2^s^−1^ (measured using a special sensor QSL-100, Biospherical Instruments Inc., San Diego, CA, USA) on a 12 h:12 h (light:dark) photoperiod. Sea anemones were fed once a week with oysters.

### 2.2. Primary Cell Cultures

From each *A. viridis* individual, an independent primary cell culture was obtained and maintained as described in Ventura et al. (2018) [15]. Briefly, cell dissociation was performed enzymatically with 0.15% collagenase type I (Sigma-Aldrich, Darmstadt, Germany). Cells were cultured at 20.0 ± 0.5 °C and in the dark, in an optimized culture medium (CM) that consisted of: 20% GMIM (Gibco, Carlsbad, CA, USA), 5% fetal bovine serum (FBS; PAA/GE Healthcare, Chicago, IL, USA), 1% kanamycin (100 μg/mL, Sigma-Aldrich), 1% amphotericin B (2.5 μg/mL; Interchim, Montluçon, France), 1% antibiotic antimycotic solution (Sigma-Aldrich), 1% L- glutamate (Sigma-Aldrich), and 71% of filtered ASW. The CM was adapted in respect to the Mediterranean Seawater characteristics (i.e., salinity 40 ppt and pH 8.1). From day 3, culture medium was replaced weekly and cells were seeded at 250,000 cells/mL in 12 well-plates.

### 2.3. Cryopreservation Protocol

As cryoprotectant, DMSO (Sigma-Aldrich) was tested at two concentrations in the final CPA solution: 5% or 10% (following Munroe et al., 2018 [33]). DMSO was dissolved in the CM or in the CM enriched with fetal bovine serum (FBS) at 25% final. Control conditions without DMSO were also tested using CM enriched or not with FBS (i.e., ‘CM’ or ‘CM + 25% FBS’).

From day 17 after dissociation, the primary cell cultures were established with reliable cellular parameters [15]. By consequence, the cultivated cells were cryopreserved at different time points, from day 17 to 45 after cell dissociation. Each cryopreserved material contained 2 million cells that were placed in a cryotube containing 1 mL of the tested solution. Cryotubes were directly placed in a −80 °C freezer (Ultra-Low Temperature VIP series, SANYO, Osaka, Japan) and kept there for 8 to 87 days.

For thawing, cryotubes were removed from the −80 °C freezer after the defined period and immediately transferred for 1–2 min into a water bath, pre-warmed at 20 °C.

For seeding the cryopreserved cells, the cryotubes were centrifuged for 5 min at 1500 rpm. The supernatant was then removed, the cell pellets resuspended in the cell culture medium and seeded at 250,000 cells/mL in 12 well-plates [15].

### 2.4. Cell Survival, Cell Viability, Cell Growth Rate, and Cell Size Assessment

Cell survival was measured right after thawing cryopreserved cells, before reseeding. It was determined as the percentage of viable cells relative to the 2 million cells initially cryopreserved. To assess the number of viable cells, a sub-sample (100 µL) of cryopreserved cells was harvested after the thawing phase. Cell viability was assessed by evaluating the membrane integrity thanks to the Evans blue method. Therefore, viable cells (unstained) and dead cells (stained) were identified and counted on a Neubauer improved hemocytometer (Sigma-Aldrich) using an optic microscope (Zeiss Axio Imager Z1).

Cell viability was measured every week to monitor the cell culture health state overtime. A sub-sample (100 µL) of cultivated cells was harvested weekly and using Evans blue method, viable cells (unstained) and dead cells (stained) were identified and counted. The cell viability was defined as the percentage of viable cells relative to total cells (i.e., viable and dead cells). In addition, two complementary methods for cell viability assessment, i.e., overall enzymatic activity using the fluorescein diacetate (FDA) staining combined with a non-vital dye (Hoechst) and cell metabolic activity with 2-(4,5-dimethyl-2-thiazolyl)-3,5-diphenyl-2H tetrazolium bromide (MTT) assay, were also conducted (see details in Supplementary Material and Methods).

Cell growth rate was also assessed every week using the previous viable cells counts with Evans blue method. The following formula was then used to calculate the 7-day averaged daily growth rate:

(1) $$\mathrm{Cell}\;\mathrm{daily}\;\mathrm{growth}\;\mathrm{rate}=\;\frac{\left({\mathrm{Viable}\;\mathrm{cells}}_{d+7}-{\mathrm{Viable}\;\mathrm{cells}}_{d}\right)/{\mathrm{Viable}\;\mathrm{cells}}_{d}}{7}\;,\;(d\;=\;\mathrm{day})$$

Cell growth rate and cell viability were monitored for each cell culture before and after cryopreservation. The monitoring of these factors for cryopreserved cells was done by considering that the age of the cells at the thawing time is the same age they were at the freezing time.

Before and after cryopreservation, during the cell counts under optic microscope (objective ×20), cells were measured, and cell sizes were scored with Zeiss microscope software Zen 2 (blue edition).

### 2.5. Hyperthermal Stress Experiment

In order to assess the maintenance of cryopreserved cells functionality, the response of cryopreserved cells to a controlled stress experiment was investigated. The cultivated *A. viridis* cells response to hyperthermal stress was assessed following the protocol published by Ventura et al. (2018) [15]. Hyperthermal stress was induced in 12-well plates exposed to two different temperatures: 20 °C (control) and 28 °C (hyperthermal condition), for 7 days. This experiment was conducted either with non-cryopreserved or cryopreserved cells, 7 days after thawing. At least four independent experiments were conducted from four primary cell cultures. For each assay, we analyzed 3 wells as technical replicates.

### 2.6. Statistical Analyses

All statistical analyses were conducted using the R v.3.6.0 software (R Foundation for Statistical Computing, Vienna, Austria) [44]. In order to assess the effect of the cryosolutions on cell survival, to compare global viability and growth between non-cryopreserved and cryopreserved cells, and to compare viability and growth rate values after hyperthermal stress, either one-way ANOVA analyses were performed when parametric analyses were possible (under normality and variance equality assumptions), or Kruskal–Wallis when non-parametric analyses were required. These analyses were followed, if necessary, by the appropriate post-hoc, i.e., Tukey for ANOVA analyses, and Dunn for Kruskal–Wallis analyses. Then, to investigate the effect of the storage duration and the cell culture age on the cell survival, correlation tests with linear regression model were conducted. Finally, repeated measures ANOVA were conducted to compare cell viability and growth through time of non-cryopreserved and cryopreserved cultures.

## 3. Results and Discussion

### 3.1. Success in Set Up of Cryopreservation Protocol on Cell Survival

**Figure 1:**
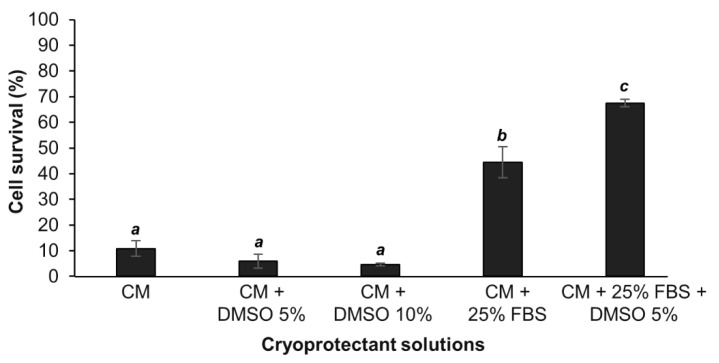
Percentage of cell survival following 10 ± 1 days of cryopreservation and thawing. For each tested cryopreservation solution, the number of viable cells were compared to the 2 million cells initially cryopreserved. Mean values and standard errors are represented, with n ≥ 3 biological replicates per condition. ANOVA revealed significant differences between data (*p* = 1.14.10^−10^) and the results from the Tukey post-hoc analysis are represented with letters: a ≠ b (*p* < 0.01), a ≠ c (*p* < 0.0001), b ≠ c (*p* < 0.05).

The efficiency of the cryopreservation solutions was first evaluated with the percentage of cells that survive a cryopreservation period of 10 ± 1 days and the subsequent thawing process. When cells were cryopreserved in the culture medium (CM) or in CM with DMSO 5 or 10% the mean percentage of cells that survived at −80 °C, was around 7%. There were no significant differences between these conditions (ANOVA; *p* > 0.05) (Figure 1). However, when cells were cryopreserved in the two conditions containing an FBS supplementation, we observed a significant higher percentage of cell survival compared to non-enriched medium conditions (*p* < 0.01 and *p* < 0.0001, respectively for the CM + 25% FBS and for the DMSO 5% in the CM+ 25%FBS). Compared to FBS enriched CM alone (±45% survival rate), adding 5% of DMSO to the FBS enriched CM significantly enhanced the survival rate of the cells to 67% (*p* < 0.05; Figure 1). Thus, the latter constituted the optimal cryosolution among those tested for cryopreserving cnidarian cells.

As it was observed in most of marine invertebrate studies [18,24,26,27,33], DMSO was found to be an efficient CPA for *A. viridis* cultivated cells when it combined with serum supplementation. The reason could be that carbohydrates, lipids and proteins present in serums act as membrane stabilizers, therefore they may help preventing membrane damage during the freezing process [45,46,47,48].

The survival rate of the designed cryopreservation protocol for *A. viridis* cultivated cells with the optimal cryosolution is comparable to those determined for dissociated cells [26,33] or other biomaterials [18] from marine invertebrates, as well as the ones from vertebrate in vitro cells [49].

**Figure 2:**
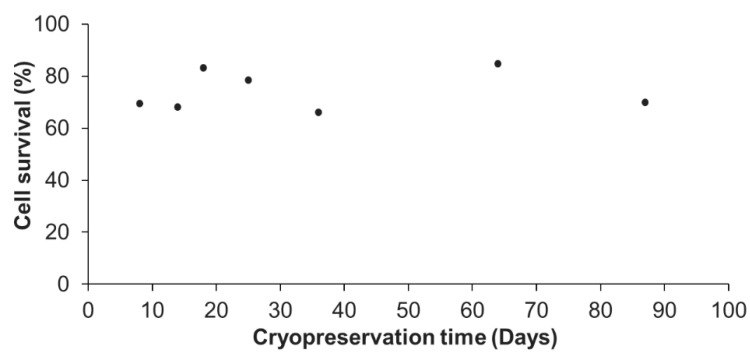
Percentage of cell survival following different cryopreservation durations and thawing. For each tested cryopreservation duration, the number of viable cells were compared to the 2 million initially cryopreserved cells. These representative results were obtained from one primary cell culture cryopreserved at 24 days after initial seeding in 7 cryotubes, each cryotube being analyzed at the end of a given cryopreservation duration (from 8 to 87 days). No significant differences between times were found (linear regression model; r^2^ = 0.037; *p* = 0.443).

Since the optimal cryopreservation solution, among those tested in this study, was found to be the DMSO 5% in FBS enriched culture medium, this solution was reused for different cryopreservation durations in order to determine the influence of the cryopreservation time on cell survival after thawing. The representative results for a *A. viridis* cell culture shown in Figure 2 demonstrated that there was no influence of the cryopreservation duration on the cell survival. Indeed, from 8 to 87 days at −80 °C, the percentage of cells that survived cryopreservation and thawing, did not vary significantly (linear regression model; coefficient not statistically significantly different from 0, *p* > 0.05). The statistical analyses done on all available data (at least 3 biological replicates) confirmed that there were no significant differences between the cryopreservation durations (*p* > 0.05; data not shown).

Cells could therefore be cryopreserved for almost 3 months without any impact on the post-thaw survival compared to short-term cryopreservation, further validating the efficiency of the designed protocol. This is a major and significant progress for marine invertebrate cell cryopreservation. In fact, although few studies evaluated the cryopreservation efficiency after a storage of several weeks [22,24,25,28] and a maximum of 12 months [30], the majority only cryopreserved cells for a few hours to a few days [23,26,27,29,33,35,36].

Cryopreservation of vertebrate cell lines is well-advanced, and protocols allow to keep cryopreserved cells for years [16]. Therefore, additional experiments are required to assess if longer cryopreservation durations are possible and/or to determine the maximal duration using the protocol developed in this study. If a duration limitation were to be found, a long-term storage in liquid nitrogen, or −150 °C freezers following the initial −80 °C freezing should be envisioned.

**Figure 3:**
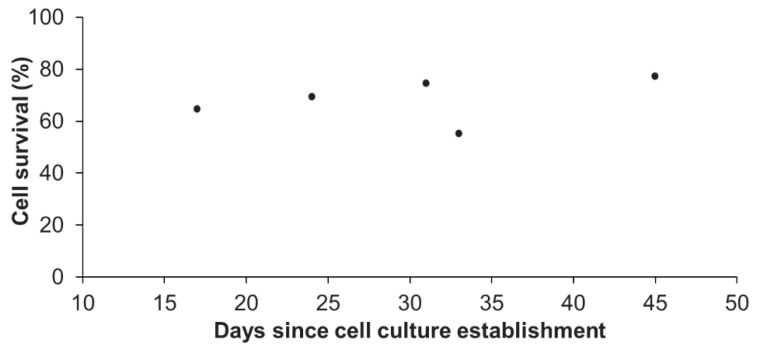
Percentage of cell survival after thawing in function of the age of the cell culture. Representative results were obtained from one primary cell culture cryopreserved at different times since its initial seeding (from day 17 to day 45). At each age tested, one cryotube was analyzed. No significant differences between ages were found (linear regression model; r^2^ = 0.15; *p* = 0.512).

In addition, we also assessed the influence of the age (time after initial seeding) of the primary cell culture on the survival at thawing. Representative results of one primary cell culture, cryopreserved at five different ages (from day 17 to day 45, Figure 3) revealed no significant differences (linear regression model; coefficient not statistically significantly different from 0, *p* > 0.05). The statistical analyses done on all available data (3 biological replicates) confirmed that there was no influence of the culture age on the cell survival (*p* > 0.05; data not shown). One primary cell culture can therefore be cryopreserved at each reseeding, as long as the cellular parameters previously defined for “healthy” *A. viridis* primary cell culture are maintained, i.e., cell aggregates formation, high viability, and constant growth rate [15]. Being able to do so, considerable amounts of cell stocks for each cell culture could be created.

### 3.2. Absence of Cryopreservation Impact on Cell Recovery and Functional Parameters

**Figure 4:**
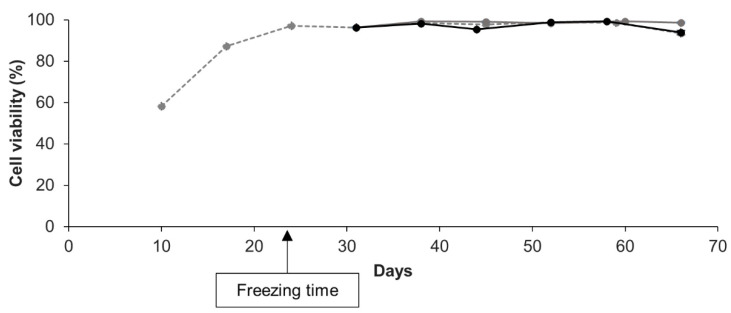
Over time, cell viability of a representative cell culture, comparing cryopreserved and non-cryopreserved cells. Non-cryopreserved culture in grey dotted line, the same culture cryopreserved at 24 days since initial seeding and thawed after 8 days of storage in grey solid line and after 36 days in black solid line. The age of the cryopreserved cells at thawing is considered the same as at the freezing time. Mean values of three technical replicates are shown with standard error bars (although not visible because smaller than the data point symbols). Repeated measures ANOVA revealed no significant differences in cell viability at each time between the non-cryopreserved culture and the cryopreserved ones (*p* = 0.582).

To assess long-term cell recovery, we measured weekly, at each reseeding, the cell viability of all primary cell cultures cryopreserved. Viability of cryopreserved cells was monitored for a period going from 6 to 12 weeks after thawing and was compared to the viability of the corresponding non-cryopreserved cell culture. Results of cell viability monitoring for 6 weeks after thawing for one primary cell culture cryopreserved at day 24 for 8 or 36 days are presented in Figure 4. The data show that cryopreserved cells were stably viable after thawing through time (>90% viability) with a cell viability equivalent to that of the corresponding non-cryopreserved cell culture (repeated measures ANOVA; *p* > 0.05). Repeated measures ANOVA conducted on all biological replicates, confirmed no differences in the cell viability over time between non-cryopreserved and cryopreserved cells nor with the cryopreservation storage period (*p* > 0.05; see Figures S1 and S2). The mean of viability, over time, was maintained at 95 ± 1.9% and at 96 ± 0.99%, respectively in the different non-cryopreserved and cryopreserved cell cultures monitored (ANOVA; *p* > 0.05; see Figure S3a). In addition, data obtained with the two complementary cell viability assays performed on non-cryopreserved and cryopreserved cells at different time points of the kinetics confirmed all these results, i.e., no differences in cell viability between non-cryopreserved and cryopreserved cells and a maintenance over time of the cell viability (ANOVA; *p* > 0.05; see Figure S4).

**Figure 5:**
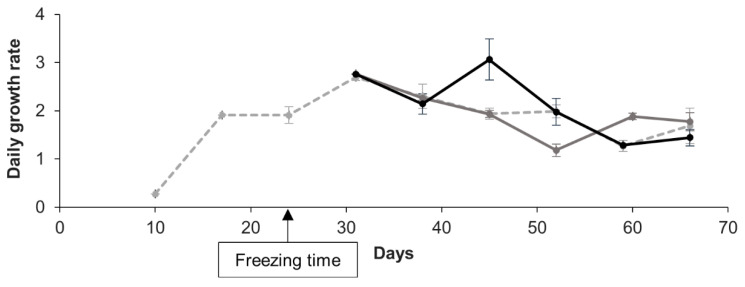
Over time, daily growth rate of a representative cell culture, comparing cryopreserved and non-cryopreserved cells. Non-cryopreserved cell culture in grey dotted line, the same culture cryopreserved at 24 days and thawed after either 8 days of storage in grey solid line or after 36 days in black solid line. Mean values of three technical replicates and standard error bars are shown. Repeated measures ANOVA revealed no significant differences in cell growth trend between the non-cryopreserved culture and the cryopreserved ones (*p* = 0.722).

As a first functional parameter, the long-term cell growth was assessed. Indeed, we considered the resumption of cell cycle after cryopreservation as an essential functional parameter to explore. Non-cryopreserved and cryopreserved cells displayed a similar daily growth rate over time and independently of the cryopreservation duration (8 or 36 days) as shown by a representative cell culture in Figure 5 (repeated measures ANOVA; *p* > 0.05).

The same result was obtained for all cell cultures tested in this study (see Figures S1 and S2). Interestingly, the cryopreserved cells stored for 79 days (Figure S2d) displayed a low initial growth rate one week after thawing and reseeding, suggesting that cryopreserved cells may need a longer time (between one and two weeks after thawing) to fully recover after such cryopreservation storage duration.

Furthermore, the mean of the daily growth rate was maintained at 1.78 ± 0.39 and at 1.73 ± 0.33, respectively in the different non-cryopreserved cell cultures and cryopreserved cell cultures monitored, with no significant differences between these two conditions (ANOVA; *p* > 0.05; see Figure S3b).

**Figure 6:**
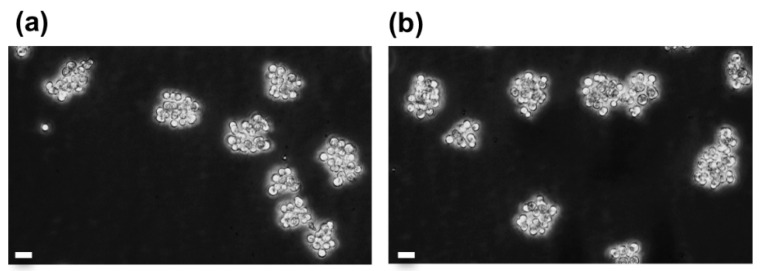
Observation of aggregates of *A. viridis* gastrodermal cells in culture before and after cryopreservation; (**a**) cell culture at day 31 since its establishment, and (**b**) the same culture cryopreserved at day 24 for 79 days, 7 days after thawing. Phase contrast microscopy (objective ×20), scale bars = 10 µm.

Long-term cell viability and growth rate monitoring of cryopreserved vs. non-cryopreserved cell cultures corroborated the healthy state of cryopreserved cells. Therefore, these analyses were completed with weekly microscope observations in order to assess the cell culture behavior. In the Figure 6, the comparison of a 31-day old non cryopreserved culture with the corresponding cryopreserved culture (considering the thawing age is equal to the freezing age) showed that cryopreserved cells, as the non-cryopreserved cells, form adherent cell aggregates, which is the characteristic architecture of *A. viridis* primary cell cultures [9,15]. In addition, cryopreserved cells in culture presented the same mean size (5.2 ± 0.49 µm) that non-cryopreserved cells (5.17 ± 0.54 µm; ANOVA; *p* = 0.957), suggesting no volume change after thawing (see Figure S5).

Therefore, cryopreserved cells maintained through time identical viability, growth, and shape to the corresponding non-cryopreserved cell culture, and this independently of the cryopreservation duration. These results indicate that the cryopreservation designed protocol stored cells in a healthy state allowing them to fully recover after thawing and to behave like their origin culture. This monitoring constitutes an essential part in validating the storage protocol for further use of cryopreserved cells and represents a major strength of this study. Indeed, the reseeding of thawed cells was rarely done on previous marine invertebrate studies, and cells are usually maintained only for a few hours to a few days [22,23,24,25,27,28], with a maximum of 15 days for bivalve cells in Dessai (2018) [30] and around 40 days for sponge cells [32,34].

**Figure 7:**
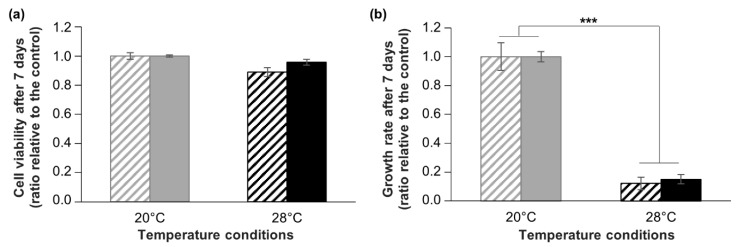
Comparison of cryopreserved and non-cryopreserved cells in response to hyperthermia. Assessment of cell viability (**a**) and growth (**b**) of *A. viridis* cells in response to a hyperthermal stress of +8 °C (black bars) for 7 days, for non-cryopreserved cultures (striped bars) and for cryopreserved cultures (filled bars). Cell viability and growth are expressed relative to control condition (20 °C; grey bars). Mean values with standard error bars are shown, biological replicates n ≥ 4. The asterisks represent the significant differences between control and stress conditions (*** Kruskall–Wallis: *p* < 0.001).

As a second parameter of the cell functionality after cryopreservation, we investigated the response of cryopreserved cells to a controlled stress experiment. In Cnidarians, and more particularly in our research model, *A. viridis*, hyperthermal stress is well known to induce oxidative damages and cell death, i.e., apoptosis [50,51,52]. Using *A. viridis* primary cell cultures, we previously reported that hyperthermia (+8 °C) did not induce any oxidative damage or impact on survival but provoked a drastic decrease of cell growth [15]. Thus, in this study, we compared the response of cryopreserved and non-cryopreserved cells submitted to the same hyperthermal stress, in terms of viability and growth. The results show that hyperthermal stress did not change significantly the cell viability neither for non-cryopreserved cells nor for cryopreserved cells (ANOVA; *p* > 0.05) (Figure 7a). Moreover, cell growth rate was drastically decreased by around 80% after 7 days at 28 °C compared to 20 °C condition (Kruskal–Wallis; *p* < 0.001), without any significant differences between non-cryopreserved and cryopreserved cells (Kruskal–Wallis; *p* > 0.05) (Figure 7b). These results are in line with data from Ventura et al. (2018) [15] and show that cryopreserved cells displayed identical resistance to non-cryopreserved cells, strongly corroborating the non-alteration of cell functionality. Therefore, not only *A. viridis* cells in vitro can be successfully cryopreserved and reseeded, but they can be reliably used for further experiments, like it is done for mammalian cells. Although functional analyses are sometimes conducted for marine invertebrate cells, through metabolic and enzymatic activities [23,24,25,26,28,30], only some sponge cells studies conducted experiments using cryopreserved cells [31,34]. However, this is a fundamental assessment in order to validate the cryopreservation protocol for creating reliable models.

## 4. Conclusions

In this study we succeeded to design an easy and rapid cryopreservation procedure for *Anemonia viridis* primary cell cultures. The established protocol enabled us to obtain high cell survival after thawing and a full long-term recovery of the cell culture behavior. The development of cryopreservation in cnidarian primary cell cultures enables us to preserve stable cell stocks available shortly after thawing for experimental procedures and sharing with the scientific community. This new tool will be an important asset to raise *A. viridis* primary cell cultures as a powerful model for studying and understanding the cnidarian properties (i.e., symbiosis lifestyle, response to stress, aging), which are difficult to study in most cnidarian models at the molecular and cellular levels.

## Supplementary Materials

The following are available online at https://www.mdpi.com/2073-4409/9/12/2541/s1, Material and Methods S1: Complementary cell viability assays; Figure S1: Over time viability (a, b, c) and daily growth rate (d, e, f) of three primary cell cultures, comparing non-cryopreserved cells (NC) and cells cryopreserved for a short storage period (C); Figure S2: Over time viability (a, b) and daily growth rate (c, d) of two primary cell cultures, comparing non-cryopreserved cells (NC) and cells cryopreserved for a long storage period (C); Figure S3: Box plots of over time viability (a) and daily growth rate (b) of all cell cultures monitored (non-cryopreserved in gray and cryopreserved in blue); Figure S4: Comparison of cell viability between 38-day old non-cryopreserved cell cultures (‘NC 38d’), and cryopreserved cell cultures, either 38-day old (‘C 38d’) or 80-day old (‘C 80d’); Figure S5: Observation, under optic microscope, of the same *A. viridis* gastrodermal cell culture (a) before and (b) after cryopreservation.
