# Supplementary material for: Cnidarian Cell Cryopreservation: A Powerful Tool for Cultivation and Functional Assays
Source: Cells. 2020 Nov 26;9(12):2541. doi: 10.3390/cells9122541 (PMC7761476; doi:10.3390/cells9122541)
Supplement: Supplementary file 1 [file cells-09-02541-s001.pdf]

Supplementary Materials

Supplementary Material and Methods

Complementary cell viability assays

FDA (Fluorescein Diacetate, 4 µg/mL; Sigma-Aldrich) and Hoechst 33342 (5 µg/mL; Sigma-Aldrich) were added and incubated with cells during 15 minutes at 20°C in the dark. Viable cells (fluorescent in green) and dead cells (fluorescent in blue) were identified and counted on a Neubauer improved haemocytometer (Sigma-Aldrich) using a fluorescence microscope (Zeiss Axio Imager Z1). The cell viability was defined as the percentage of viable cells relative to total cells (i.e. viable and dead cells).

MTT assay was performed following manufacturer instructions with slight modifications. Briefly, prior the assay 60 000 cells were seeded in triplicate in 96-well plate in 100 µL of culture medium and incubated for 24h. 20 µL of 5mg/mL MTT solution (Sigma-Aldrich) is then added to each well and incubated 5h at 20°C in the dark. Then, the supernatant is removed, and the yielded formazan was dissolved in the suitable detergent (isopropanol) for 15 minutes. Subsequently, the plates' light absorption (OD) is read at wavelength 590 nm on spectrofluorometer (SAFAS, Monaco). The cell viability was expressed as follow: Viability % = (cryopreserved cells OD /non-cryopreserved cells OD) ×100

Supplementary Figures

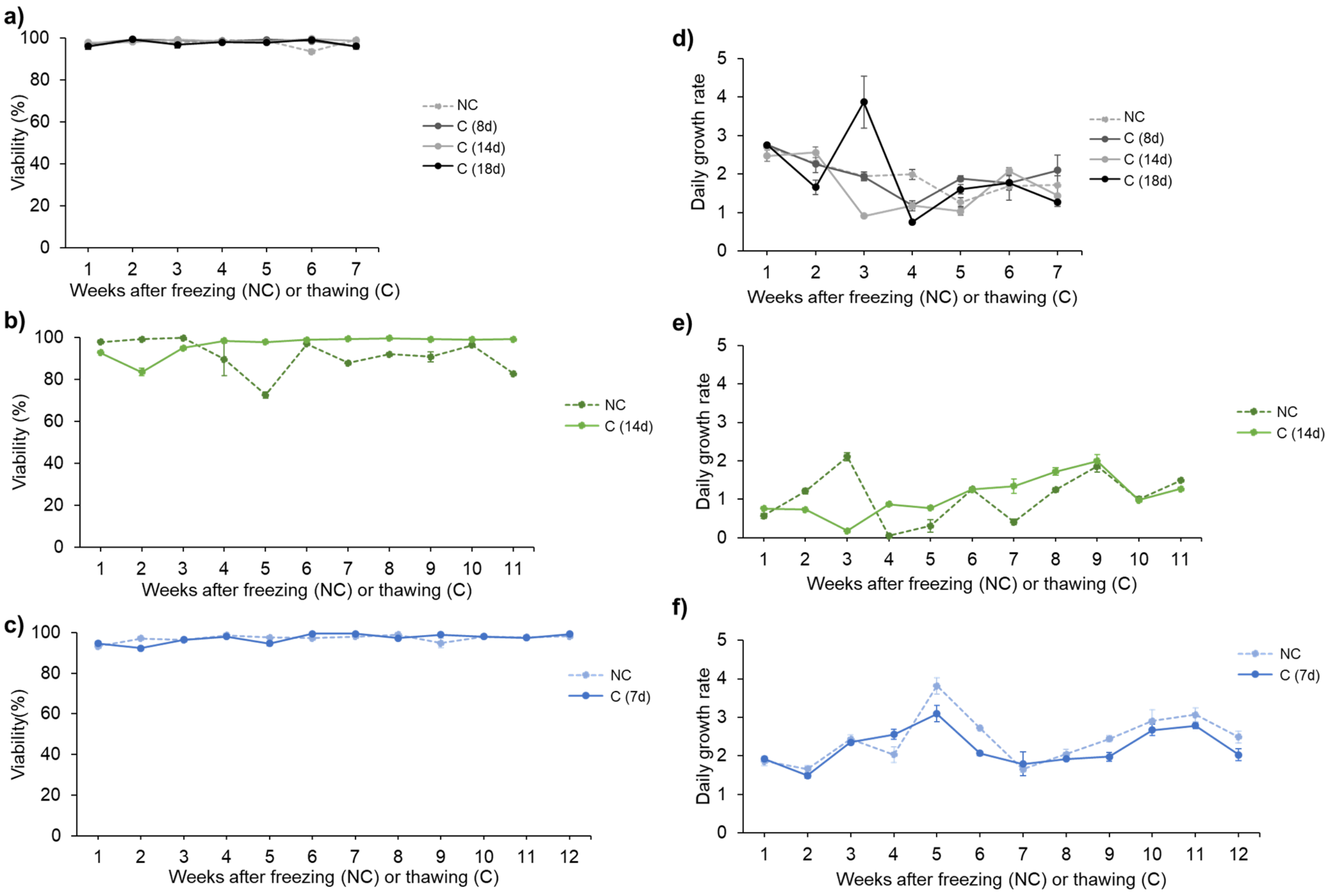

Figure S1. Over time viability (**a, b, c**) and daily growth rate (**d, e, f**) of three primary cell cultures, comparing non-cryopreserved cells (NC) and cells cryopreserved for a short storage period (C). The cryopreservation storage period is mentioned in the legend, it goes from 8 days ('8d') to 18 days ('18d'). Each graph per variable measured represent one primary cell culture and its corresponding cryopreserved cell culture(s). Time points are mentioned as "Week after freezing (NC) or thawing (C) time" i.e. the monitoring presented here for the non-cryopreserved cultures begins after the freezing

time, and the one for cryopreserved cells is done after thawing, thus considering the age at that time is the same age as at the freezing time. .

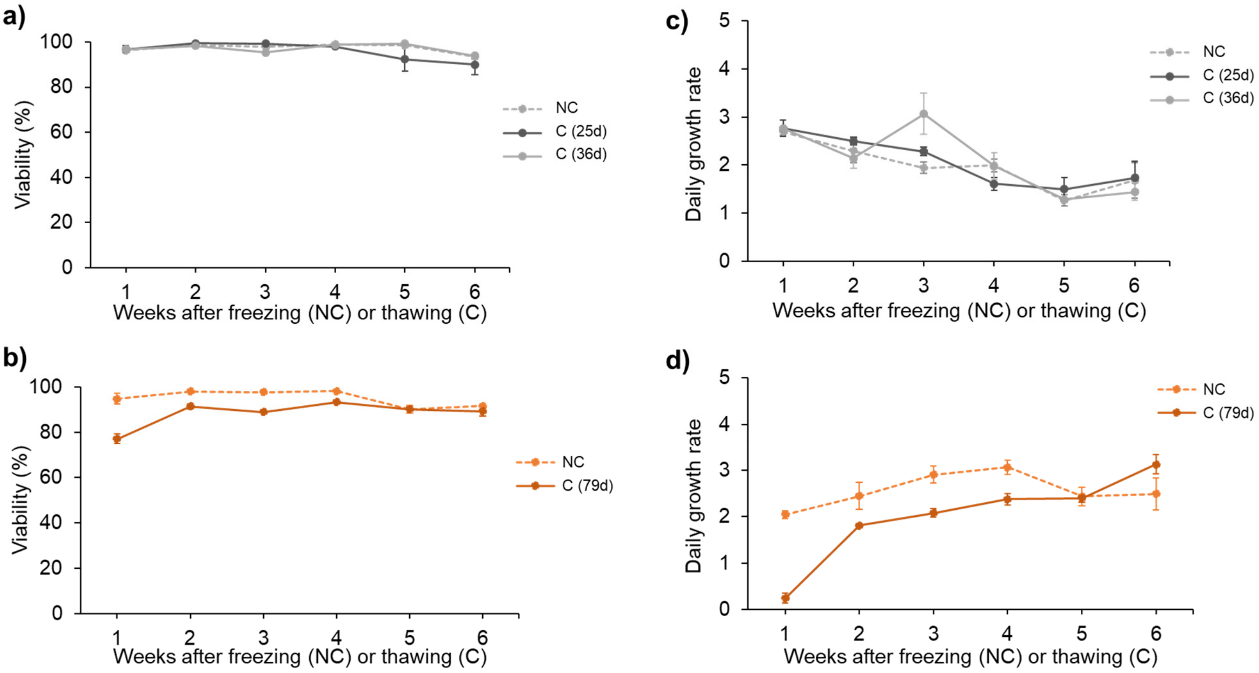

Figure S2. Over time viability (**a, b**) and daily growth rate (**c, d**) of two primary cell cultures, comparing non-cryopreserved cells (NC) and cells cryopreserved for a long storage period (C). The cryopreservation storage period is mentioned in the legend, it goes from 25 days ('25d') to 79 days ('79d'). Each graph per variable measured represent one primary cell culture and its corresponding cryopreserved cell culture(s). Time points are mentioned as "Week after freezing (NC) or thawing (C) time" i.e. the monitoring presented here for the non-cryopreserved cultures begins after the freezing time, and the one for cryopreserved cells is done after thawing, thus considering the age at that time is the same age as at the freezing time.

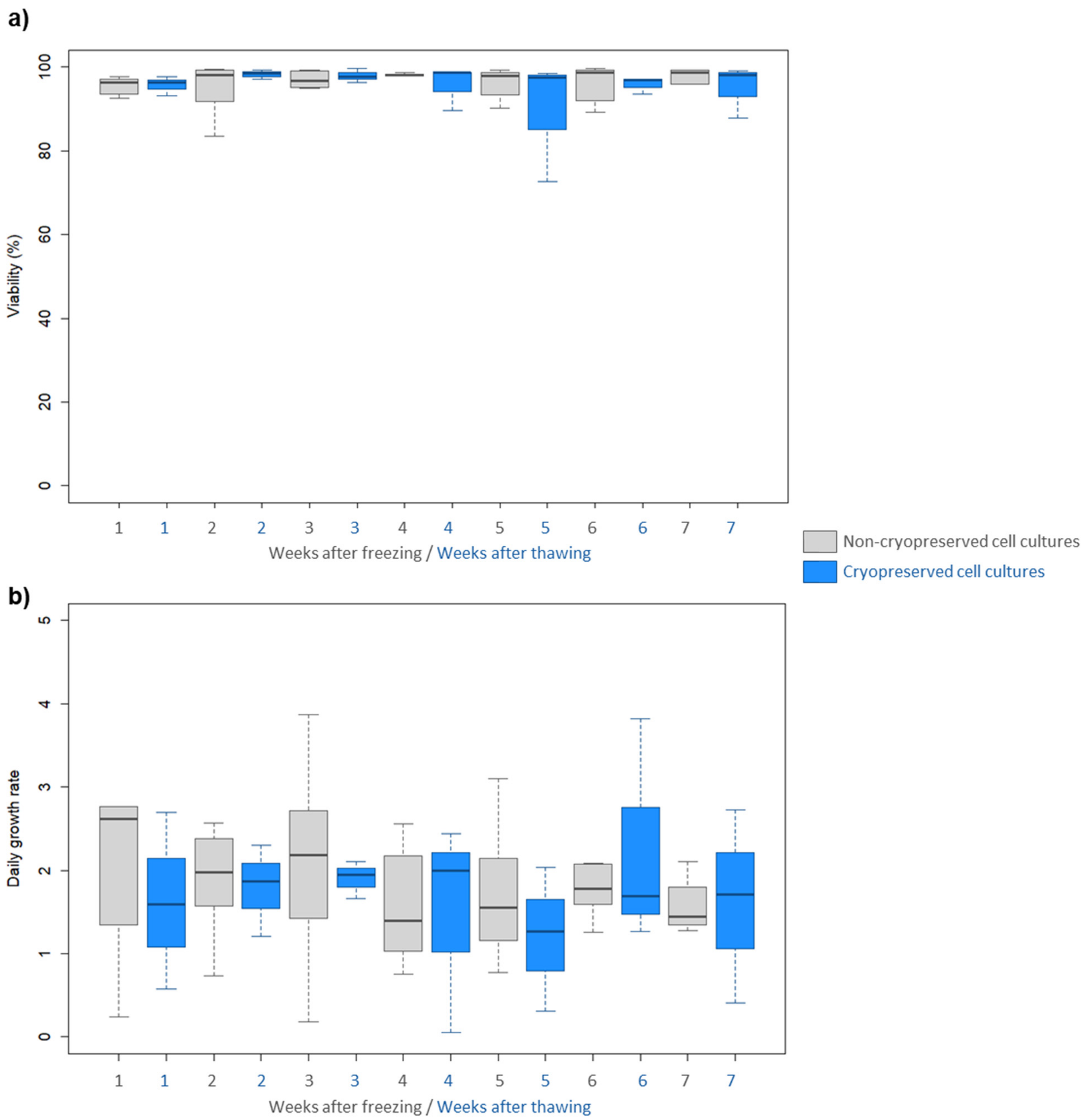

Figure S3. Box plots of over time viability (**a**) and daily growth rate (**b**) of all cell cultures monitored (non-cryopreserved in gray and cryopreserved in blue). Time points are identical to the ones present in Fig. S1 and S2 and are here going from 1 to 7 weeks, which represent the time points after freezing (for non-cryopreserved cells) or after thawing (for cryopreserved cells) where at least 3 biological replicates were monitored.

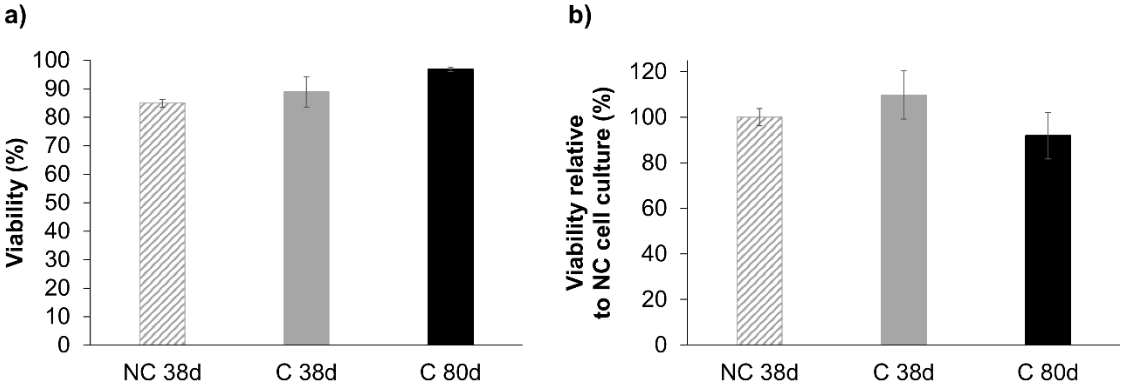

Figure S4. Comparison of cell viability between 38-day old non-cryopreserved cell cultures ('NC 38d'), and cryopreserved cell cultures, either 38-day old ('C 38d') or 80-day old ('C 80d'). Cell viability was measured with (**a**) FDA/Hoechst staining and with (**b**) MTT assay. Mean values with standard error bars are shown (n≥3). ANOVA analyses revealed no significant differences in the viability values between non-cryopreserved and cryopreserved cells, and over time between cryopreserved cells (p=0.45 for panel a; p=0.378 for panel b).

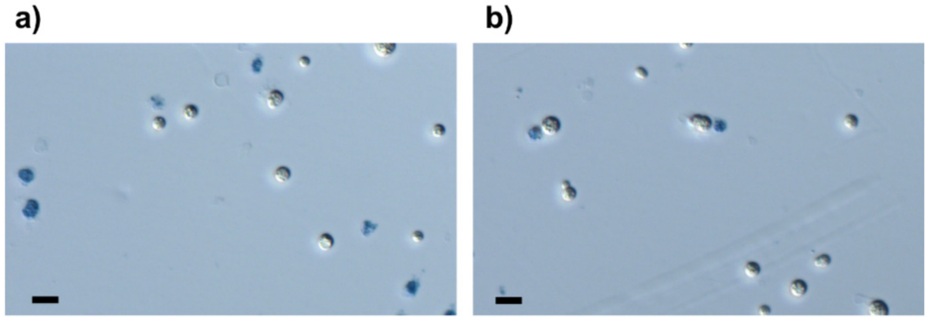

Figure 5. Observation, on Neubauer improved haemocytometer under optic microscope (objective x20), of the same *A. viridis* gastrodermal cell culture **(a)** before and **(b)** after cryopreservation and stained with Evans blue (i.e. the dead cells stained in blue) (scale bar = 10 μm).
